# A Novel Natural Penetration Enhancer for Transdermal Drug Delivery: In Vitro/In Vivo Evaluation and Penetration Enhancement Mechanism

**DOI:** 10.3390/pharmaceutics17020254

**Published:** 2025-02-14

**Authors:** Nanxi Zhao, Jiale Hao, Yucong Zhao, Bingqian Zhao, Jiayu Lin, Jian Song, Manli Wang, Zheng Luo

**Affiliations:** 1 Department of Pharmaceutical Sciences, College of Pharmacy, Beihua University, Jilin 132013, China; nancy_z3023@126.com (N.Z.);; 2 Department of Pathology, Jilin Central Hospital, Jilin 132013, China

**Keywords:** penetration enhancer, essential oil, perilla ketone, penetration enhancement mechanism, transdermal drug delivery

## Abstract

**Objectives**: This study aimed to identify and develop a novel, safe, and effective transdermal penetration enhancer derived from the leaves of *Perilla frutescens* (L.) Britt, and to explore the underlying mechanisms of its penetration enhancement effects. **Methods**: To evaluate the safety profile of the penetration enhancer, both skin irritation tests and histopathological analyses were conducted. The transdermal enhancement capabilities of the penetration enhancer were assessed in vitro using five model drugs. Furthermore, to gain insights into the penetration enhancement mechanism of this novel penetration enhancer, a range of analytical methods were used, including a spectroscopic technique, differential scanning calorimetry, micro-optical techniques, and molecular docking simulations. **Results**: Perilla essential oil contained 93.70% perilla ketone (PEK), which exhibited a safety profile superior to that of azone. PEK significantly increased the cumulative skin permeation of all the model drugs (*p* < 0.05). PEK exhibited the most obvious impact on puerarin penetration, with quantitative enhancement ratios of 2.96 ± 0.07 and 3.39 ± 0.21 at concentrations of 3% and 5% (*w*/*v*), respectively. A strong correlation between the enhancement effect of PEK and the physicochemical properties of the drugs was observed. Mechanistic studies revealed that PEK facilitates drug distribution from the solution phase to the stratum corneum (SC). **Conclusions**: PEK, seldom discussed in former studies, was observed to show extensive penetration enhancement effects by inducing conformational changes in SC lipids and disrupting the tightly ordered bilayer arrangement of lipids. These findings highlight the potential of PEK as a promising and safe natural transdermal penetration enhancer.

## 1. Introduction

Transdermal drug delivery systems (TDDSs) involve delivering drugs through the skin at a fixed rate into the systemic circulation, serving as an alternative to oral and intravenous administration [1]. Unlike traditional administration methods, TDDSs circumvent first-pass metabolism in the liver, improving bioavailability, maintaining stable blood drug concentrations, and effectively reducing drugs’ toxicity and side effects [2]. However, the stratum corneum (SC) on the outermost layer of the skin—characterized by a tightly arranged “brick and mortar” structure—acts as a significant barrier to transdermal drug absorption [3]. Researchers have explored various methods over the past four decades to overcome the SC’s barrier function, with penetration enhancers emerging as a cost-effective and flexible solution for improving transdermal drug absorption [4].

Recent work has demonstrated the limitations of chemical penetration enhancers, including toxicity and skin irritation [5]. As a representative of traditional chemical penetration enhancers, an ethanol-based system was associated with skin irritation or contact dermatitis [6], while azone showed significantly higher toxicity compared with plant-derived essential oils [7]. After application to the skin, essential oils and their components could be rapidly metabolized, without accumulating in the organism and quickly excreted, strongly indicating that they are safe [8]. Consequently, plant-derived essential oils and their active ingredients have gained considerable attention due to their safety profiles [9]. For instance, nifedipine transdermal patches formulated with clove essential oil as a penetration enhancer demonstrated superior in vitro permeation [10]. Similarly, 1,8-cineole from eucalyptus oil enhanced chlorhexidine retention in the skin [11]. Essential oils from *Epilobium angustifolium* L., rich in α-caryophyllene oxide, eucalyptol, β-linalool, camphor, (S)-carvone, and β-caryophyllene, increased the cumulative skin permeation of ibuprofen and lidocaine by 1.32 and 1.23 times, respectively [12]. These active ingredients, predominantly terpenes and monoterpenes, enhance transdermal permeation by interacting with intercellular lipids in the SC [12,13]. Perilla essential oil (PO), extracted from the leaves of *Perilla frutescens* (L.) Britt, has shown potential as a transdermal penetration enhancer [14]. However, its actual efficacy and underlying mechanisms remain unverified. Penetration enhancers exhibit drug specificity; for example, polyglyceryl-3 dioleate is more effective for drugs with lower polar surface areas (PSAs) and polarizability [15], while borneol promotes the permeation of drugs with lower log *P* values and higher molecular weights [16]. Additionally, the polarity and hydrogen bonding ability of drugs significantly influence their transdermal permeation [17]. Understanding the differential enhancement effects of penetration enhancers on drugs with varying physicochemical properties is crucial for optimizing TDDS formulations.

**Figure 1:**
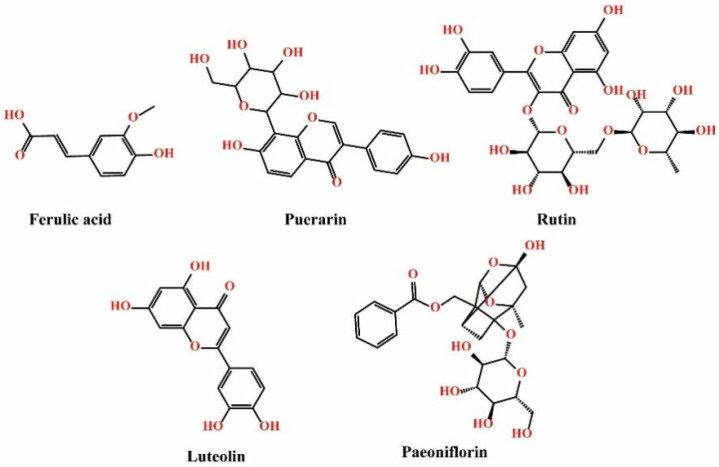
Chemical structures of the model drugs.

**Figure 2:**
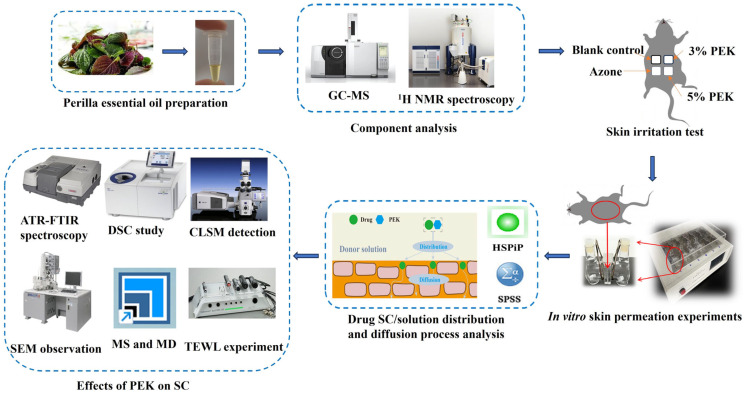
Flowchart for the PO preparation and evaluation processes. (GC-MS: gas chromatography–mass spectrometry; ^1^H NMR: proton nuclear magnetic resonance; PEK: perilla ketone; SC: stratum corneum; ATR-FTIR: attenuated total reflectance–Fourier transform infrared spectroscopy; DSC: differential scanning calorimetry; CLSM: confocal laser scanning microscope; SEM: scanning electron microscope; MS: molecular dynamics simulation; MD: molecular docking; TEWL: transepidermal water loss).

The purposes of this study were to develop a novel, safe, and effective transdermal penetration enhancer derived from the leaves of *Perilla frutescens* (L.) Britt, verify its penetration enhancement effects, and investigate the underlying mechanisms from a comprehensive, multifaceted perspective. The essential oil extracted from *P. frutescens* leaves was analyzed using gas chromatography–mass spectrometry (GC-MS), and the molecular structures of its main components were verified using proton nuclear magnetic resonance (^1^H NMR). Penetration-enhancing effects were assessed through an in vitro skin permeation test; the correlations between the drug’s skin permeation-related parameters and the drug’s physicochemical properties were acknowledged. Five model drugs with varying physicochemical parameters, including peoniflorin, luteolin, rutin, ferulic acid, and puerarin, were selected to evaluate their penetration enhancement effects. These drugs were chosen based on their PSAs, hydrogen bond (H-bond) acceptors, and H-bond donors. Their chemical structures are shown in Figure 1, and their physicochemical properties are detailed in Table 1. To ensure the safety and application potential of PO in TDDSs, in vivo skin irritation tests were conducted. The penetration enhancement mechanism was systematically evaluated using attenuated total reflectance–Fourier transform infrared spectroscopy (ATR-FTIR), differential scanning calorimetry (DSC), a confocal laser scanning microscope (CLSM), a scanning electron microscope (SEM), molecular docking (MD), molecular dynamics simulation (MS), and transepidermal water loss (TEWL) tests. The preparation and evaluation processes for PO are depicted in Figure 2.

## 2. Materials and Methods

### 2.1. Materials and Animals

Fresh perilla leaves identified as *Perilla frutescens* (L.) Britt were collected in Jilin, China. Peoniflorin was purchased from Xi’an Virgin Biotechnology Co., Ltd. (Xi’an, China). Luteolin, rutin, ferulic acid, puerarin, isopropyl palmitate (IPP), perilla ketone (PEK), hematoxylin and eosin (H&E), and fluorescein isothiocyanate (FITC) were purchased from Shanghai Macklin Biochemical Technology Co., Ltd. (Shanghai, China). All the other chemicals and solvents used in this study were reagent grade and obtained commercially.

Wistar rats (male; weight: 180–220 g; age: eight weeks) were supplied by Yisi Experimental Animal Technology Co., Ltd. (Changchun, China; license number: SCXK (Ji) 2020-0002). All the animals were housed with free access to food and water under a 12:12 h light–dark cycle at 25 ± 2 °C. Animals were anesthetized by intraperitoneally injecting a 20% (*w*/*w*) urethane solution before the experiment. The experiments were conducted in accordance with the Guide for the Care and Use of Laboratory Animals, and the animal study was approved by the Animal Ethics Committee of Beihua University (2024082206). Many drug molecules are significantly more permeable through the rat epidermis compared to the human epidermis [18]. Rat models present the disadvantage of an extremely high density of hair follicles, meaning that the hair needs to be removed. While human skin is more appropriate for evaluating the efficacy of topical and transdermal drug delivery systems, its availability is limited, so rat skin is often utilized as a substitute [18,19]. Porcine skin closely resembles human skin due to similarities in various properties such as thickness, hair density, and epidermal turnover time [18]. However, conducting studies with porcine skin in vivo can be labor-intensive and costly. Consequently, rat skin was employed as an animal model in vitro and in vivo in this study.

### 2.2. Preparation and Determination of Perilla Essential Oil

Fresh perilla leaves were cut into small pieces of approximately 1 cm^2^ and placed in a 5000 mL round-bottom flask, into which distilled water was added at a 1:10 ratio (*w*/*w*). The flask was sealed with a piece of plastic wrap and kept in a cool place for 3 h, allowing the perilla leaf pieces to soak thoroughly. The flask was then connected to an essential oil extraction device, and 1 mL of ether was used as the extractant. The solution in the flask was heated and kept boiling; a condensation reflux rate of 80 drops per minute was maintained. The heating was performed continuously for 3 h, after which the solution was cooled to room temperature. The organic layer was collected, and the aqueous layer was extracted thrice with ether. The organic layers were merged and dehydrated with anhydrous sodium sulfate, after which the ether in it was evaporated, and the container was sealed and stored in a refrigerator at 4 °C.

The components of PO were analyzed using the GCMS-TQ8040 NX (Shimadzu Co., Kyoto, Japan). GC conditions: a TG-5M (30 m × 0.25 mm × 0.25 μm) capillary chromatography column. Heating program: starting at 60 °C, heating up to 280 °C at 10 °C·min^−1^, and maintaining for 5 min. Injection port temperature: 280 °C. Flow rate: 12 mL·min^−1^. Injection mode: split injection. Split ratio: 10:1. Split duration: 0.8 min. Peak pressure: 5.00 kPa. MS conditions: ion source, EI source; voltage, 70 eV; ion source temperature, 250 °C; solution delay, 3 min; scanning range, 30–500 amu; stay scanning, 0.2 min.

Owing to the relatively single and large proportion of principal components for PO, AVANCE 500 MHz ^1^H NMR spectroscopy (Bruker, Fallanden, Switzerland) was used for the further chemical structure verification of the principal components, and MestReNova 9.0 software was applied for data processing.

### 2.3. Skin Irritation Test

Skin irritation refers to reversible changes that occur locally on the skin after the application of a test substance. The skin irritation response is mainly characterized by the activation of T-cells and can induce symptoms on the skin surface such as erythema and edema [20]. The degree to which the penetration enhancers irritated the skin was analyzed using a self-contrasted method. The rats were anesthetized by intraperitoneally injecting 20% urethane. The hair on the surface of the abdominal skin was removed, and the undamaged skin selected was divided into three areas of 1 cm × 1 cm each; one was set as the blank control, one was treated with azone as the positive control, and one was treated with PEK. The skin surface was cleaned with normal saline before the experiment. The administration area was coated with 10 μL of the test samples after the skin was completely dried and covered with double-layer gauze, and the gauze was fixed with medical anti-allergic tape. The erythema index (*EI*) of the abdominal skin surface of rats was detected using the Mexamter MX18 pigment testing probe in the Cutometer^®^ Dual MPA580 system (Courage+Khazaka Electronic GmbH, Cologne, Germany) at 0 min, 15 min, 30 min, 1 h, 2 h, 4 h, 6 h, 8 h, 24 h, and 36 h after administration. The testing probe measured the red pigment on the skin surface based on optical principles, and the *EI* value could be directly read from the software of the system. The higher the value, the greater the degree of erythema on the skin surface. Another group of rats was treated using the above-mentioned methods. The experimental rats were anesthetized and euthanized when the *EI* value was at its maximum. The skin was removed and soaked in 4% paraformaldehyde for its fixation, embedded in paraffin, sectioned, and then stained using H&E. The stained sections were then observed under an optical microscope.

### 2.4. In Vitro Permeation Study

#### 2.4.1. Preparation of Isolated Skin

The skin was prepared according to a previous study [21]. Rats were anesthetized through the intraperitoneal injection of 2% urethane. The hair on the surface of the abdominal skin was removed. The rats were sacrificed; then, undamaged skin from the rat abdomen was selected, and the subcutaneous fat and mucosa were removed. The skin samples were stored at −80 °C and used within one week.

#### 2.4.2. Drug Solubility in Donor Solutions

The drug solubility in the donor solution (*C*) was assessed. An excess amount of the model drug was added to IPP with or without PEK. The mixture was shaken vigorously for 48 h in a water bath at 32 °C and then centrifuged. The supernatant was analyzed using HPLC after appropriate dilution. The LC-2010A HT liquid chromatograph (Shimadzu Corporation, Kyoto, Japan) with a C18 column (200 mm × 4.6 mm, 5 μm) was used. The chromatographic conditions for the model drugs are shown in Table S1, and the flow rate was set at 1 mL·min^−1^.

#### 2.4.3. In Vitro Skin Permeation Experiments

The impact of PEK on the permeation of model drugs was assessed through in vitro skin permeation experiments. Horizontal dual-chamber diffusion cells, each with an effective diffusion area of 1.77 cm^2^, were used for the studies [22]. Rat abdominal skin was isolated and mounted between the two chambers. IPP served as the donor vehicle [23], and the donor solutions were prepared by dissolving varying amounts of model drugs in IPP, with and without PEK. In the preliminary experiments, PEK produced better penetration-enhancing effects at concentrations of 3% and 5% (*w*/*v*) than at the other concentrations; thus, 3% and 5% (*w*/*v*) PEK were included in the formal experiments. Different receptor solutions were used for the different drugs: a pH 7.4 phosphate-buffer solution (PBS) was used for ferulic acid, peoniflorin, and puerarin, while pH 7.4 PBS containing 30% (*w*/*v*) PEG 400 was used for luteolin and rutin. At the start of the experiment, 4 mL volumes of both the donor and receptor solutions were placed in the respective chambers, which were maintained at 32 °C with constant rotation at 600 rpm [24]. At predetermined time intervals (1, 2, 3, 4, 5, 6, 7, and 8 h), 2 mL samples of the receptor solution were withdrawn for HPLC analysis. The chromatographic conditions for the model drugs are shown in Table S1. Each sample was replaced with an equivalent volume of fresh receptor solution to maintain sink conditions. Each experiment was repeated four times for consistency.

#### 2.4.4. Data Evaluation

The cumulative skin permeation of the model drugs was calculated based on the concentrations and volumes of the receptor solutions at each sampling time point, as represented by the following equation:

(1) $$Q=\left(4C_{i}+\sum_{i=1}^{n-1}2C_{i-1}\right)/1.77$$

where *Q* is the cumulative amount of the drug permeated and *C_i_* is the concentration in the receiver chamber at time point *i*.

To assess the penetration enhancement capacity of PEK, the quantitative enhancement ratio (*QER*) was calculated as shown in Equation (2). The cumulative amount per unit area was plotted against time, and the steady-state flux (*J*_ss_) was calculated from the slope of the linear portion of the plot. The lag time (*T*_lag_) was determined from the point at which the linear portion intersected the *x*-axis [25].

*QER* = *Q*_with PEK_/*Q*_without PEK_ (2)

The drug permeability coefficient (*P*) and enhancement ratio (*PER*) of the drug permeability coefficient were calculated as follows [26]:

*P* = *J*_ss_/*C* (3)

*PER* = *P*_with PEK_/*P*_without PEK_ (4)

where *C* is the drug solubility in the donor solution.

### 2.5. SC/Solution Partition of Drugs

To study the distribution of drugs between the SC and the solution, rat abdominal skin was treated at 37 °C with pH 7.4 PBS containing 0.4% trypsin for 24 h [27]. After treatment, the SC was carefully separated using a cotton swab, cleaned with pure water, and vacuum-dried. A 10 mg sample of the SC was then incubated in an IPP solution (with or without PEK) containing the model drug at 32 °C for 24 h. Following incubation, the SC was removed, cleaned, and cut into pieces. These pieces were then placed in methanol, and the solution was sonicated for 30 min and centrifuged to obtain the supernatant. The concentration of the drug in both the SC and the solution was measured using HPLC, with four parallel trials conducted for each group. The SC/solution partition coefficient (*K*) for each drug was calculated, along with the partition enhancement ratio (*KER*) for drug distribution to the SC, using the following formulas:

*K = C*_SC_/*C*_vehicle_ (5)

*KER* = *K*_with PEK_/*K*_without PEK_ (6)

### 2.6. Solubility Parameter Calculation

The solubility parameter (*δ*) is determined by the individual dispersion, polar, and H-bond solubility parameters, as described previously [28]. The *δ* values for the model drugs, PEK, IPP, and ceramide NP (NP) were calculated using HSPiP 6.0 software (Steven Abbott, Horsholm, Denmark). Ceramides are key components of intercellular lipids, and NP plays a crucial role in skin barrier function [29], with a significant presence in both human and rodent skin [30]. Consequently, NP was chosen as the representative of intercellular lipids.

### 2.7. Correlation Analysis

To explore the impact of distribution and diffusion processes on the cumulative permeation of drugs, a correlation analysis was performed between *KER*, *PER*, and *QER*. Additionally, the relationships between *KER*, *PER*, and the physicochemical properties of the drugs were examined to assess the influence of PEK. Pearson correlation coefficients were calculated to confirm the strengths of the relationships, and linear or multilinear regressions were conducted using SPSS 29.0 (IBM Co., New York, NY, USA).

### 2.8. Identification of the Effects of Perilla Ketone on Stratum Corneum

#### 2.8.1. Attenuated Total Reflectance–Fourier Transform Infrared Spectroscopy

ATR-FTIR spectroscopy was used to assess changes in the organization of skin lipids following the addition of PEK [31]. The rat skin was mounted between two vertical diffusion chambers. A 200 μL sample of the donor solution was applied to the SC side, while 4 mL of PBS was used as the receptor medium. After 4 h, the skin was removed from the diffusion cell, and its surface was gently wiped with cotton. The skin was then positioned with the SC side facing down on a ZnSe crystal and analyzed in the wavenumber range of 4000–400 cm^−1^ using an IR spectrometer (NEXUS + 70, Thermo Electron Corporation, Waltham, MA, USA).

#### 2.8.2. Differential Scanning Calorimetry Study

The skin treatment procedure followed the method outlined in Section 2.5. The SC was immersed in an ethanol solution containing varying concentrations of PEK at room temperature for 0.5 h, after which the ethanol was removed through vacuum drying. A 5 mg piece of the SC (either treated or untreated) was cut into small segments and placed in an aluminum pan. All the samples were analyzed using a Mettler-Toledo thermal analyzer (DSC1, Mettler-Toledo International Inc., Zurich, Switzerland) with a temperature range of 0–120 °C and a heating rate of 5 °C·min^−1^ under nitrogen flow [27].

#### 2.8.3. Confocal Laser Scanning Microscope Detection

CLSM was used to visualize the skin enhancement effect of PEK, using FITC as the fluorescent probe. FITC and PEK were mixed into IPP and sonicated until the FITC was completely dissolved. The supernatant was used as the donor solution, and FITC in IPP without PEK was used for the control group. The rat skin was mounted between two vertical diffusion chambers, and the prepared solution was applied to the skin for 4 h. Afterward, the skin was rinsed with distilled water and gently dried with filter paper to remove the excess solution on its surface [17]. The skin was then placed on a glass slide, and CLSM analysis was conducted using an LSM 710 Laser Scan Microscope (Carl Zeiss AG, Jena, Germany). The excitation wavelength was set to 520 nm, with a scanning depth of 0–20 μm and a step length of 4 μm.

#### 2.8.4. Scanning Electron Microscope Observation

SEM was used to observe the effect of PEK on the structure of the SC of the skin. In vivo rat abdominal skin samples were prepared using the method described in Section 2.4.1. A 3% or 5% (*w*/*v*) PEK solution was applied to the surface of the skin samples, respectively. After a 4 h treatment with PEK, the samples were fixed with 2.5% glutaraldehyde at 4 °C for 24 h. The samples were then rinsed three times with 0.1 M PBS (pH = 7.2) and fixed with a 1% osmium tetroxide solution for 2 h. Following another three rinses with 0.1 M PBS, the samples were dehydrated using ethanol solutions. The skin samples were then dried, and platinum metal was sprayed onto the surfaces of the samples. The samples were observed, and images were obtained using a Hitachi SU8010 SEM (Hitachi High Technologies Co., Tokyo, Japan).

#### 2.8.5. Molecular Docking

MD was used to investigate the interaction between the model drugs, PEK, and NP. This study used Materials Studio version 7.0 software (Accelrys Inc., San Diego, CA, USA) [32]. The chemical structures of the compounds were optimized using the Forcite module, and MD was performed in the Blend module. The lowest energy conformation of the mixture under the COMPASS II force field was calculated, and the docking energy was determined using the Dreiding force field [33].

#### 2.8.6. Molecular Dynamics Simulation

MS was conducted to further examine the interactions between lipids and the components in PO, as well as the dynamic behavior of the chemicals. NP, representing intercellular lipids, was used to create a lipid box in the Amorphous Cell module of Materials Studio software 7.0 (Accelrys Inc., San Diego, CA, USA). The chemicals were placed in the module according to the proportions of the actual formulations, which were optimized using the Universal module. NVT (30 ps) and NPT (300 ps) calculations were performed at 305 K with an initial time step of 5 ps and a total simulation length of 200 ps [24]. The cohesive energy density (*CED*) was determined for each system, and the diffusion coefficient (*D′*) was calculated from the mean square displacement (MSD) curve using the Einstein equation.

#### 2.8.7. Transepidermal Water Loss Experiment

TEWL experiments were conducted using methods outlined in the literature [34]. The rat abdominal skin was prepared as described in Section 2.3, but the skin was divided into four areas. One area served as a blank control, one area was treated with 5% (*w*/*v*) azone, and two areas were treated with 3% and 5% (*w*/*v*) PEK, respectively. The TEWL values of rat abdominal skin were measured at the designated time points using the Tewameter TM HEX Moisture Loss Probe in a Cutometer^®^ Dual MPA580 system (Courage+Khazaka Electronic GmbH, Cologne, Germany).

### 2.9. Statistical Analysis

Experimental data are expressed as the means ± standard deviations (SDs). Statistical analysis was performed using SPSS 29.0 software (IBM Co., New York, NY, USA), and the significance level was set at *p* < 0.05. All the data were checked for normality and homoscedasticity before regression analysis.

## 3. Results and Discussion

### 3.1. Preparation and Determination of Perilla Essential Oil

**Figure 3:**
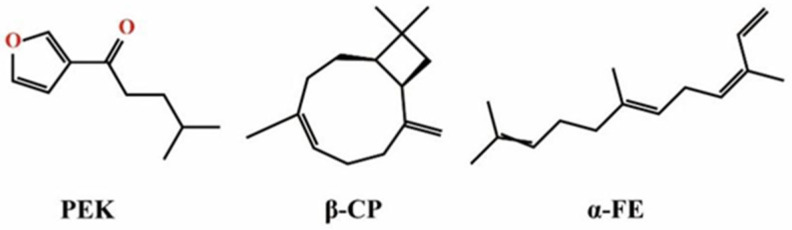
Chemical structures of the three compounds in PO. (PO: perilla essential oil; PEK: perilla ketone; β-CP: β-caryophyllene; α-FE: (*Z*,*E*)-α-farnesene).

PO extracted through steam distillation is a light-yellow liquid. GC-MS analysis was used to identify the components of PO from *P. frutescens* [35]. In total, 23 components were identified in the essential oil (the GC chromatogram is shown in Figure S1, and the components of PO are listed in Table S2). Notably, the content of PEK, the main component, was found to be as high as 93.70%. Only two other compounds, β-caryophyllene (β-CP, 2.30%) and (*Z*,*E*)-α-farnesene (α-FE, 1.03%), had concentrations exceeding 1%. The molecular structure of the main component in PO was further confirmed using ^1^H NMR, with the following results: ^1^H NMR (CDCl_3_) δ: 0.87 (d, 6H, 2CH_3_), 1.45 (m, 1H, CH), 1.54 (m, 2H, CH_3_), 2.81 (t, 2H, CH_3_), 7.13 (d, H, CHO), 7.16 (d, H, CH), and 8.61 (s, H, CHO). The hydrogen spectrum data were in line with those reported in other studies [36], confirming that PEK is the main component of PO. The structures of these three compounds are shown in Figure 3. According to the literature, the PO obtained in this study was derived from PK-type perilla leaves, which are widely distributed in nature and represent an easily obtainable plant resource. The PEK in PO is a monoterpene compound, while β-CP and α-FE are terpene compounds commonly found in plant essential oils. All three components have the potential to enhance penetration based on the classification of transdermal penetration enhancers [5]. However, due to the exceptionally high content of PEK in PO, which far exceeds the other components, PEK is likely to play a crucial role in enhancing drug skin permeation and was therefore selected for further investigation in this study.

### 3.2. Skin Irritation Test

**Figure 4:**
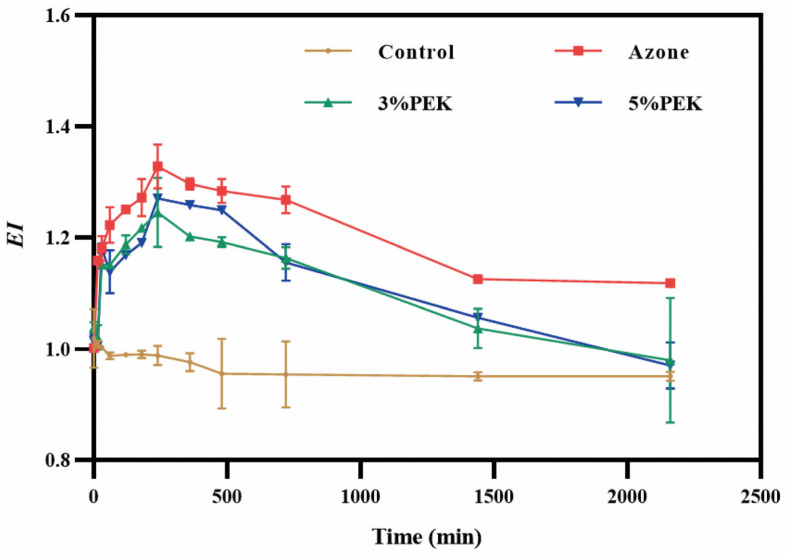
Dynamic skin irritation caused by penetration enhancers (*n* = 4, *EI*: erythema index; PEK: perilla ketone).

To assess the potential of the penetration enhancers to irritate the skin, a non-invasive in vivo skin erythema measurement was performed. The profile of the *EI* values following the topical administration of the penetration enhancers is shown in Figure 4. The results indicated that the *EI* values in the azone group were significantly higher than those in the other groups (*p* < 0.05) and remained elevated, not returning to baseline levels until 36 h after treatment. Due to its potent skin irritation, azone is often used as a positive control in studies evaluating the skin irritation potential of plant essential oils and their components as penetration enhancers [37]. Furthermore, azone can cause severe skin allergic reactions and edema that are irreversible [38]. In contrast, the maximum skin irritation response induced by PEK occurred at 4 h, with the *EI* value nearly returning to its baseline after 24 h.

**Figure 5:**
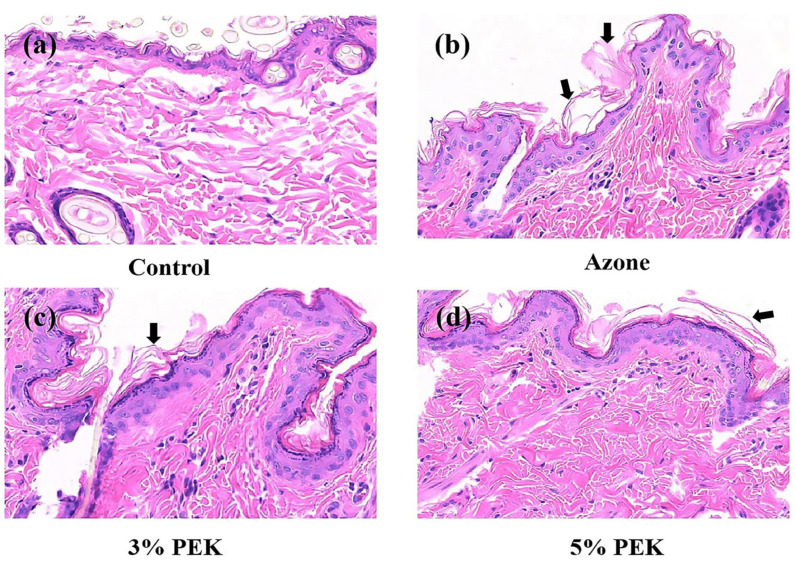
Histological pictures of the skin treated with penetration enhancers. (**a**) Skin without treatment. (**b**) Skin treated with azone. (**c**) Skin treated with 3% PEK (*w*/*v*). (**d**) Skin treated with 5% PEK (*w*/*v*). (H&E staining: 200×; PEK: perilla ketone).

The histopathological results are shown in Figure 5. As shown in Figure 5, in the control group, the skin tissue’s structure appeared normal, with clear epidermal, dermal, and subcutaneous layers. No hyperkeratosis or abnormalities in the SC were observed, and there was no degeneration, necrosis, or pathological changes such as congestion or edema in the dermis. Additionally, no abnormalities were observed in accessory structures, such as hair follicles or glands. After treatment with azone, the SC was thickened, and areas of separation within the SC were observed. In contrast, following PEK treatment, only mild separation of the SC was observed in certain areas, with no abnormalities in other tissue structures. These results from the *EI* value detection and histopathological analysis indicate that PEK, as a natural terpenoid compound, is safer than azone.

### 3.3. In Vitro Permeation Study

#### 3.3.1. Data Evaluation

**Figure 6:**
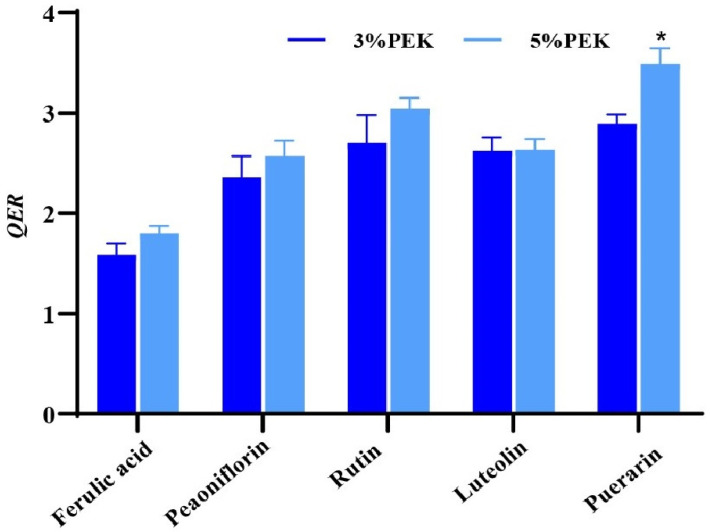
Enhancement effects of PEK on different model drugs (mean ± SD, *n* = 4; * *p* < 0.05 compared to the 3% PEK group; *QER*: quantitative enhancement ratio; PEK: perilla ketone).

Currently, there are no published reports regarding the use of PEK as a penetration enhancer. This study evaluated the effects of PEK at concentrations of 3% and 5% (*w*/*v*) in IPP on the skin permeation of five model drugs, with the detailed results shown in Table 2. The data indicated no consistent pattern regarding the influence of PEK on *T*_lag_. The *J*_ss_ and *Q* values for the five model drugs all exhibited increases under the influence of PEK, with the effects showing a concentration-dependent trend. The *QER* for the model drugs, shown in Figure 6, further highlights that PEK at different concentrations can effectively enhance the percutaneous absorption of these drugs to varying degrees. Meanwhile, analysis of the *K* values revealed that PEK significantly facilitated the transfer of drugs from the donor solution into the SC (the *KER* is shown in Table S3). Additionally, both concentrations of PEK notably enhanced the permeation of the model drugs (the *PER* is shown in Table S4).

#### 3.3.2. Correlation Analysis

To better understand the impact of the drug SC/solution distribution and diffusion processes on the overall permeation behavior, and to investigate the enhancement effect of PEK, a multiple linear regression analysis was performed on the *QER*, *KER*, and *PER* values. The regression equation for PEK at a concentration of 3% (*w*/*v*) was as follows:

*QER* = 0.434 *KER* + 0.346 *PER* + 0.703 (*r* = 0.995, *p* = 0.003) (7)

The regression equation for PEK at the concentration of 5% (*w*/*v*) was as follows:

*QER* = 0.422 *KER* + 0.350 *PER* + 0.758 (*r* = 0.882, *p* = 0.005) (8)

**Figure 7:**
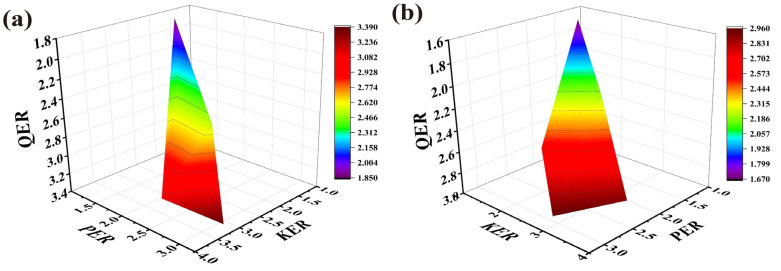
Response surface plot illustrating the effects of *KER* and *PER* on *QER*. (**a**) The perilla ketone concentration was 3% (*w*/*v*). (**b**) The perilla ketone concentration was 5% (*w*/*v*). (*QER*: quantitative enhancement ratio; *PER*: enhancement ratio of the drug permeability coefficient; *KER*: partition enhancement ratio for drug distribution to the stratum corneum).

The regression curves are presented in Figure 7. The results indicate a positive correlation between the *QER* and both the *KER* and *PER*, suggesting that PEK enhances cumulative transdermal permeation by facilitating both the distribution and diffusion of the drugs.

##### Effects of PEK on the Distribution of Drugs with Different Physicochemical Properties in the SC

Analysis of the relationship between the *KER* value and the physicochemical properties of the drugs revealed a correlation between the *KER* value and PSA and the number of hydrogen bond donors of the drugs. The regression equation for PEK at a concentration of 3% (*w*/*v*) was as follows:

*KER* = 0.011 *PSA* + 0.234 H-bond donor + 1.987 (*r* = 0.907, *p* = 0.001) (9)

The regression equation for PEK at a concentration of 5% (*w*/*v*) was as follows:

*KER* = 0.015 *PSA* + 0.314 H-bond donor + 1.042 (*r* = 0.761, *p* = 0.009) (10)

**Figure 8:**
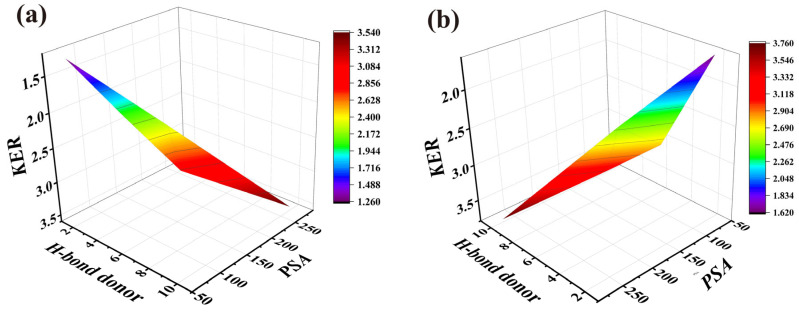
Response surface plot illustrating the effects of PSA and H-bond donor on *KER*. (**a**) The perilla ketone concentration was 3% (*w*/*v*). (**b**) The perilla ketone concentration was 5% (*w*/*v*). (*PSA*: polar surface area; *KER*: partition enhancement ratio for drug distribution to the stratum corneum).

Equations (9) and (10) demonstrate that the *KER* value is positively correlated with the PSA and H-bond donor number of the drug. The regression curves are presented in Figure 8. These findings suggest that the formation of intermolecular H-bonds plays a crucial role in the ability of the penetration enhancer to promote the drug’s distribution from the solution to the SC [39]. When the number of H-bond donors in the drug’s molecular structure increased, its ability to form intermolecular H-bonds was enhanced, making the process of distribution from the solution to the SC more sensitive to regulation by PEK. This could be attributed to the fact that PEK contains only H-bond acceptors; PEK can act as an H-bond receptor, forming complexes with the drugs. This interaction reduces the overall polarity of the complex and facilitates the drug’s distribution into the SC [40]. For instance, rutin, which contains 10 H-bond donors in its structure and has the highest number of H-bond donors among the model drugs, exhibited the highest *KER* value.

##### Effects of Perilla Ketone on the Diffusion of Drugs with Different Physicochemical Properties in the SC

The analysis of the relationship between the *PER* value and the physicochemical properties of the drugs showed a positive correlation between the *PER* value and the difference in solubility parameters (∆*δ*) between the model drug and NP. The regression equation for PEK at a concentration of 3% (*w*/*v*) was as follows:

*PER* = 0.058 ∆*δ* + 1.103, *r* = 0.803 (11)

The regression equation for PEK at a concentration of 5% (*w*/*v*) was calculated as follows:

*PER* = 0.064 ∆*δ* + 1.119, *r* = 0.854 (12)

**Figure 9:**
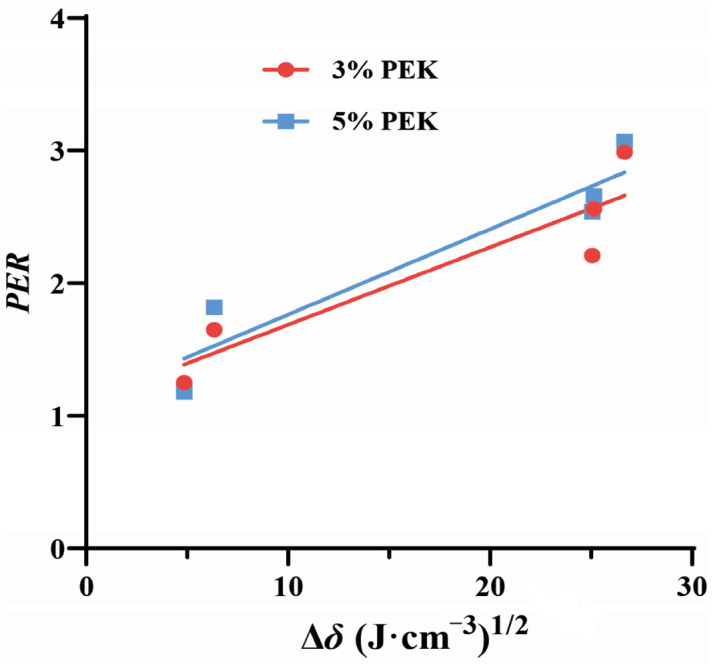
The correlation between the *PER* and ∆*δ*. (*PER*: enhancement ratio of the drug permeability coefficient; PEK: perilla ketone; Δ*δ*: the difference in solubility parameters).

Hypothetically, the larger the ∆*δ* value, the smaller the interaction between substances and the poorer their compatibility [41]. The δ values of the model drugs, PEK, and NP, as well as the ∆*δ* values between them, are shown in Table 3. The linear relationship described by Equations (11) and (12) was well-established, signifying that, as the ∆*δ* value between the drugs and NP increases, the penetration enhancement ability of PEK also increases. The correlation curves are shown in Figure 9.

The permeability coefficient can be expressed as the ratio of the product of the diffusion coefficient and the *K* to the skin thickness [42]. As discussed previously, the formation of H-bonds facilitates the distribution of drugs into the SC. However, the speed of the drug’s diffusion from the skin into the receptor solution is influenced by the drug’s inherent properties, such as its molecular weight, p*K*_a_, and solubility. The solubility parameter is a comprehensive reflection of a compound’s dispersion force, polarity, and hydrogen bonding ability. Therefore, the correlation between the solubility parameters and the *PER* values was examined in this study. As shown in Table 3, the solubility parameter of PEK was very close to that of NP (the main lipid in the SC) and significantly lower than that of the model drugs. This suggests that PEK has a stronger affinity for NP than the model drugs. Consequently, PEK tends to interact with intercellular lipids in the SC, disrupting the ordered structure of the lipid bilayer and promoting drug permeation [43]. Moreover, the disruptive effect of penetration enhancers on lipids is correlated with the formation of intermolecular H-bonds [15]. Therefore, based on the in vitro drug skin permeation data from this study, it is hypothesized that PEK can act as an H-bond receptor, forming intermolecular H-bonds with lipids. This interaction may hinder the formation of H-bonds between lipids and drugs to some extent, thereby promoting the diffusion of drugs in the SC.

**Figure 10:**
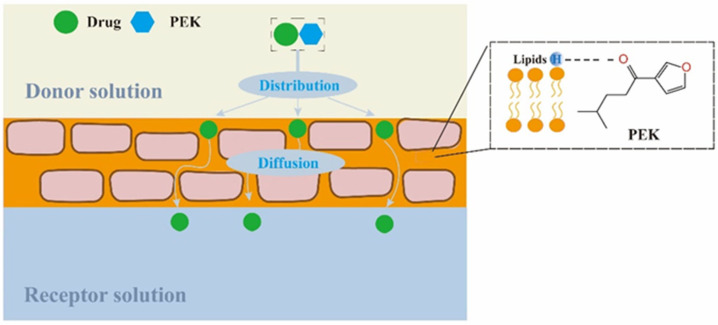
Mechanism by which PEK enhances a drug’s skin permeation (PEK: perilla ketone).

Through analysis of the SC/solution distribution and diffusion processes for the model drugs, it can be inferred that PEK primarily enhances penetration by forming intermolecular H-bonds with lipids in the SC, which interferes with the binding of drugs to the lipids (Figure 10). However, its penetration enhancement ability may still be limited by the structure and physicochemical properties of the drug itself. The key findings can be summarized as follows: PEK was the main component of PO and did not cause any skin irritation; PEK notably enhanced the skin permeation of all the model drugs; PEK might interact with lipids in the SC, enhancing SC/solution distribution and diffusion processes for the model drugs.

The SC is the main barrier structure in the skin, and PEK’s ability to disrupt this structure could accelerate the diffusion of drug molecules in the SC. Further research is needed to explore the interaction between PEK and the SC at the molecular level.

### 3.4. Effects of Perilla Ketone on Stratum Corneum

#### 3.4.1. Attenuated Total Reflectance–Fourier Transform Infrared Spectroscopy Analysis

**Figure 11:**
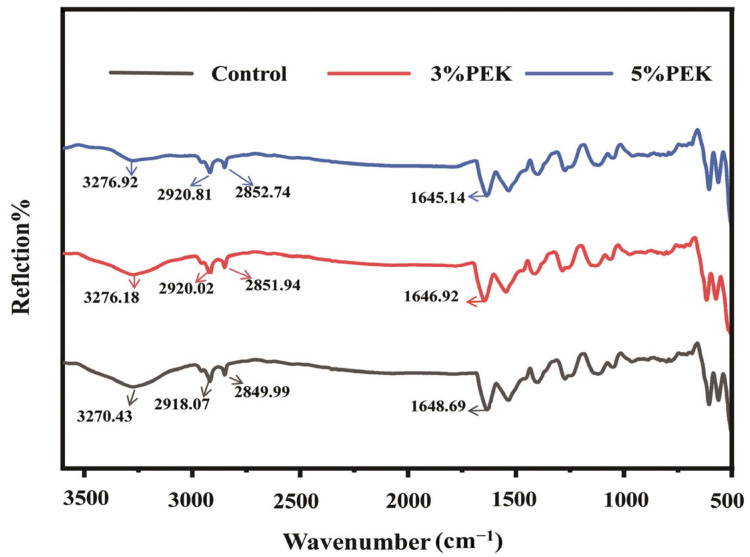
ATR-FTIR spectra with or without PEK (PEK: perilla ketone).

ATR-FTIR analysis of the skin was performed to investigate changes in the functional groups of the SC [44] and provide further insights into the mechanism by which PEK enhances transdermal drug delivery. As described in the literature, SC lipids exhibit characteristic peaks for the CH_2_ asymmetric and symmetric stretching vibrations at approximately 2920 cm^−1^ and 2850 cm^−1^, respectively [45]. In the control group, the C-H stretching absorption peaks were observed at 2918.07 cm^−1^ (ν_as_CH_2_) and 2849.99 cm^−1^ (ν_s_CH_2_). After treatment with PEK, the CH_2_ absorption peaks shifted toward higher wavenumbers, the peak areas decreased, and the degree of change increased with higher PEK concentrations, as shown in Figure 11 and Table 4. The shift in the CH_2_ peak toward higher wavenumbers indicates a change in the alkane chain conformation of the lipids from a trans to a gauche form, leading to a reduction in the ordered arrangement of lipid bilayers [46]. The extent of the shift in C-H stretching vibration is related to the trans/gauche conformer ratio in the alkyl chain [47]. A higher displacement of the C-H stretching vibration suggests stronger interference with lipid side chains, greater lipid mobility, and, consequently, a more pronounced enhancement effect of the PEK [48]. Additionally, because the areas of the two CH_2_ peaks are proportional to the lipid content in the SC, the decreased peak areas imply lipid extraction by the PEK [49]. Overall, the CH_2_ asymmetric and symmetric stretching vibration data indicate that PEK can effectively disrupt the tight arrangement of lipid bilayers, extract lipids from the SC, and accelerate the diffusion of drug molecules in a concentration-dependent manner.

The H-bonds between the head groups of SC lipids in the opposing lamellae are crucial for bilayer structure and barrier function [50]. Under normal conditions, ceramides, which make up the largest proportion of SC lipids, are tightly packed in the bilayer due to extensive hydrogen bonding. Adjacent ceramides form H-bonds via their amide groups with the hydrophilic heads, creating a robust network. If penetration enhancers possess stronger hydrogen bonding capabilities, they can disrupt this tight hydrogen bond network, facilitating transdermal drug absorption [51]. The displacement of the Amide I band shifted from 1648.69 cm^−1^ in the control group to 1646.92 cm^−1^ and 1645.14 cm^−1^ in the low- and high-concentration PEK-treated groups, respectively. This shift to higher wavenumbers indicates that PEK forms H-bonds with the amide groups in the hydrophilic head of ceramides after entering the SC, leading to the disruption of the lipid bilayer’s H-bond network [52]. The intermolecular H-bonds between PEK and lipids hinder the formation of H-bonds between lipids and drugs, accelerating the diffusion of drugs in the SC. Compared with those in the control group, the vibration absorption peaks of the OH group in the two PEK-treated groups also shifted to higher wavenumbers, indicating hydration [53]. Hydration enhances the hydrophilicity of the SC, which is beneficial for the distribution of highly polar drugs from the solution to the SC.

In summary, PEK forms intermolecular H-bonds with the hydrophilic head of SC lipids, altering the conformation of lipid chains. This enhances the fluidity of the lipid bilayer, thereby promoting drug transdermal permeability through its combined effects on SC hydration and lipid extraction. The increased drug crossing the epidermis can be absorbed through the capillaries in the dermis to increase the amount of drug entering circulation. By promoting the transdermal absorption of drugs, penetration enhancers are expected to improve the bioavailability of drugs in TDDSs, which has been demonstrated in some clinical trials [54].

#### 3.4.2. Differential Scanning Calorimetry Study

**Figure 12:**
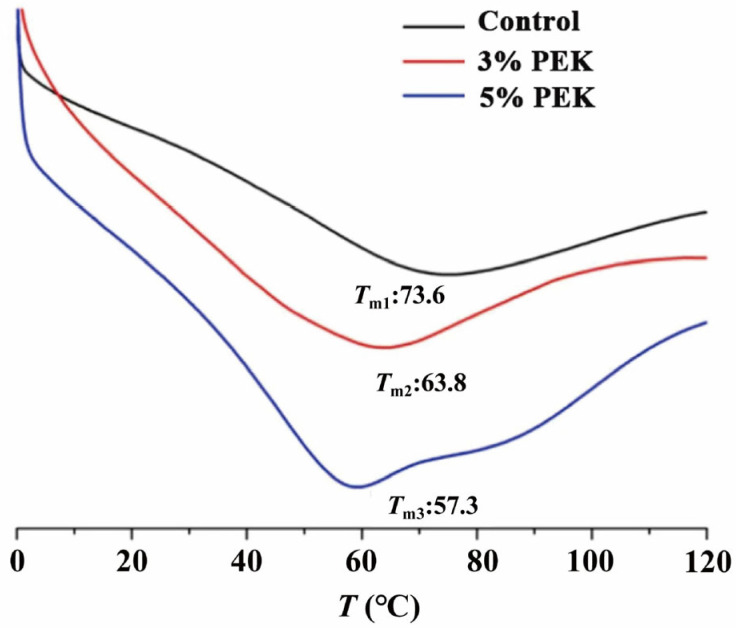
DSC curve of the SC with or without PEK (*T*_m_: transition temperature; PEK: perilla ketone).

A DSC study was conducted to examine the endothermic transition peaks of the SC. The transition temperatures (*T*_m_) are shown in Figure 12. After treatment with low and high concentrations of PEK, the Tm values of the skin decreased from 73.6 °C in the control group to 63.8 °C and 57.6 °C, respectively. The enthalpy change (Δ*H*) values for the untreated SC, 3% PEK-treated SC, and 5% PEK-treated SC were 1.76, 2.12, and 2.20 kJ·mol^− 1^, respectively. The Δ*H* values also decreased by 12.39% and 36.10%, respectively. These changes are attributed to the disruption of the ordered arrangement of intercellular lipid bilayers and the subsequent enhancement of bilayer fluidity [55]. The DSC data indicate that PEK promotes disorder within the lipid bilayers, facilitating the transition of lipid–keratin complexes from a gel to a liquid state [56]. As the PEK concentration increases, its effect on lipids intensifies, reducing the resistance to drug diffusion within the SC. This increases the skin permeation of the model drugs as the PEK concentration rises. These findings are consistent with the ATR-FTIR results.

#### 3.4.3. Confocal Laser Scanning Microscope Detection

**Figure 13:**
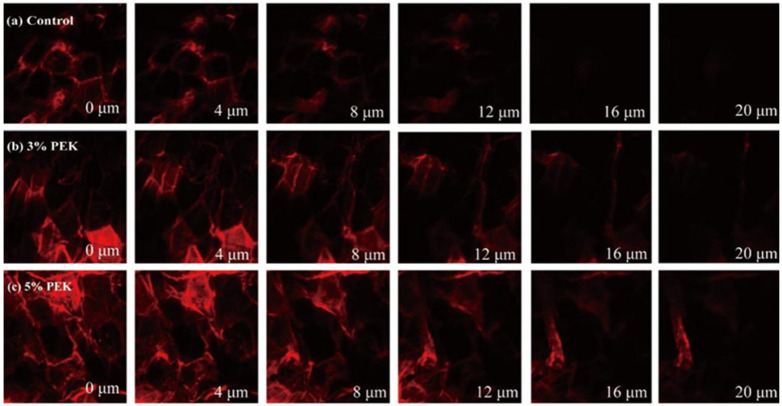
CLSM optical images of the skin at different depths after treatment with PEK. (**a**) Skin without treatment. (**b**) Skin treated with 3% PEK (*w*/*v*). (**c**) Skin treated with 5% PEK (*w*/*v*). (PEK: perilla ketone).

A CLSM was used to visualize the transdermal permeation of drugs, employing FITC as a fluorescent probe to explore the mechanism by which PEK enhanced penetration [57]. Optical images of the skin are shown in Figure 13, which reveal flat, hexagonal keratinocytes on the skin surface, and the transdermal permeability pathway of FITC was clearly intercellular, involving the lipid pathway. In the control group, the cell morphology remained intact, and the maximum penetration of FITC into the skin was 12 μm. After treating the skin with 3% (*w*/*v*) PEK, the penetration depth of FITC increased to 16 μm, and the fluorescence intensity indicated a significant increase in the concentration of FITC in the SC. When the PEK concentration increased to 5% (*w*/*v*), the penetration depth of FITC further increased to 20 μm, with a higher fluorescence intensity than that observed with the lower PEK concentration. Moreover, compared with those in the control group, the keratinocytes in the PEK-treated groups showed deformed and irregular morphology, further supporting the hypothesis that PEK disrupts the ordered arrangement of intercellular lipids, potentially causing keratinocytes to undergo morphological changes due to compression [58]. The in vitro skin permeation study using FITC as a surrogate for model drugs clearly demonstrated that PEK effectively increased the concentration of drugs in the SC and facilitated their diffusion.

#### 3.4.4. Scanning Electron Microscope Observation

**Figure 14:**
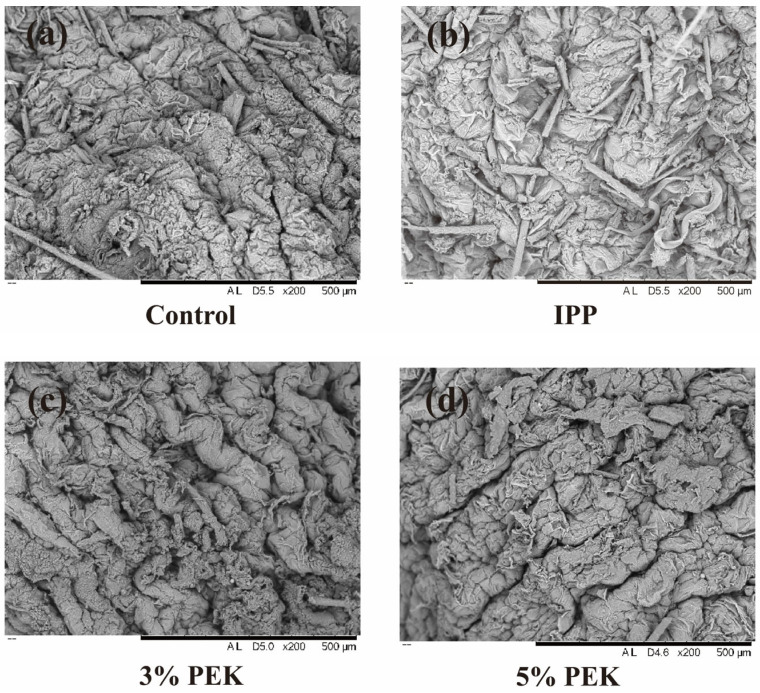
Microstructure of the rat skin’s SC after treatment with PEK. (**a**) Skin without treatment. (**b**) Skin treated with donor vehicle IPP. (**c**) Skin treated with 3% PEK (*w*/*v*). (**d**) Skin treated with 5% PEK (*w*/*v*). (IPP: isopropyl palmitate; PEK: perilla ketone).

An SEM was used to monitor the morphological changes in the skin [52]. The surface of the normal rat skin’s SC appeared relatively flat, with minimal folds, hair, and skin appendages. The keratinocytes were arranged in a regular, neat, and tightly stacked layered structure, contributing to the skin’s barrier function, as shown in Figure 14a. The changes in the microstructure of the rat skin’s SC after treatment are depicted in Figure 14b–d. Compared with that for normal skin, the SC in the solvent IPP group showed no significant changes, maintaining a layered and ordered stacking arrangement. However, in the 3% (*w*/*v*) PEK-treated group, a certain degree of loosening in the tightly stacked layers was observed, with significantly deeper and more pronounced folds on the surface and small amounts of keratinized scales flipping up. The effect was even more pronounced in the 5% (*w*/*v*) PEK-treated group, where small cracks and local shedding of the SC were observed. The loosening of the SC can be attributed to the lipid-disrupting effect of PEK, while the increase in skin wrinkles may result from hydration. The upward movement and shedding of keratin scales were likely due to the lipid extraction effect of PEK. SEM observations revealed the microscopic changes in the SC, supporting the hypothesis that PEK enhances the distribution and diffusion of drugs in the SC. Additionally, the concentration-dependent penetration enhancement effect of PEK on drugs was evident.

#### 3.4.5. Molecular Docking

**Figure 15:**
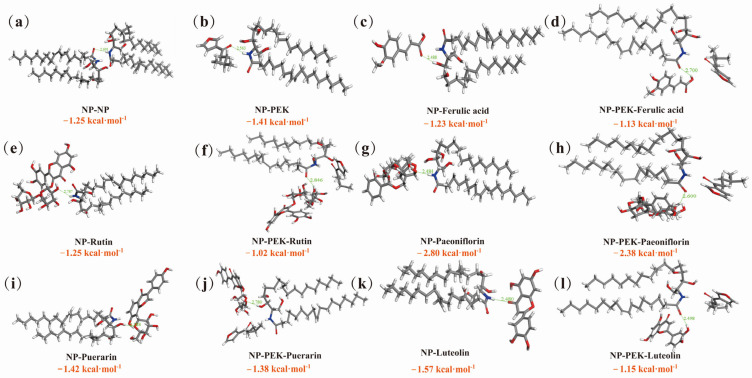
Snapshots of the optimal binding sites for hydrogen bonds between each group of molecules. (**a**) Binding site between NP and NP. (**b**) Binding site between NP and PEK. (**c**) Binding site between NP and ferulic acid. (**d**) Binding site between NP-PEK and ferulic acid. (**e**) Binding site between NP and rutin. (**f**) Binding site between NP-PEK and rutin. (**g**) Binding site between NP and paeoniflorin. (**h**) Binding site between NP-PEK and paeoniflorin. (**i**) Binding site between NP and puerarin. (**j**) Binding site between NP-PEK and puerarin. (**k**) Binding site between NP and luteolin. (**l**) Binding site between NP-PEK and luteolin. (Gray: carbon; red: oxygen; blue: nitrogen; white: hydrogen. H-bonds are presented as light green dotted lines; H-bond energy values are described in orange letters; NP: ceramide NP; PEK: perilla ketone).

In the MD study, NP was chosen as a substitute for SC lipids. MD was performed on NP-NP, NP-PEK, and NP-model drugs (with or without PEK). The docking energy of the H-bonds between each group of molecules was negative, with bond lengths less than 3.4 Å, suggesting that hydrogen bonds are more likely to form between these molecules. A larger absolute bond energy value indicates stronger hydrogen bonding and tighter binding between the molecules [59]. Snapshots of the optimal binding sites for H-bonds between each group of molecules are shown in Figure 15, with the corresponding distances between two atoms labeled. The carbonyl group in PEK acted as an H-bond receptor, forming intermolecular H-bonds with the amide group of the hydrophilic head of NP. Compared with that of the intermolecular H-bonds in NP-NP, the bond length was shorter, and the bond energy was larger. These results further corroborate the ATR-FTIR data, suggesting that PEK breaks the H-bond network of ceramides by forming more stable intermolecular H-bonds with NP. The reason for the intermolecular competitive hydrogen bonding may be that the side chains of PEK are significantly shorter than those of ceramides, giving PEK a structural advantage that facilitates its insertion into the ordered lipid bilayers. In addition, the ketocarbonyl and furan in PEK molecules form an unsaturated conjugated system, which has a strong ability to attract electrons, further promotes the formation of intermolecular competitive H-bonds, and eventually loosens the tight H-bond network formed at the head of ceramides. The intermolecular competitive hydrogen bonding disrupts the ordered lipid bilayers, reducing the barrier function of the SC and enhancing drug diffusion. In the absence of PEK, the model drugs served as H-bond receptors, forming intermolecular H-bonds with NP. The optimal binding site for NP with ferulic acid and puerarin was the hydroxyl group of the hydrophilic head, while for peoniflorin, luteolin, and rutin, it was the amide group. When PEK was introduced into the system, the H-bond binding energy between each model drug and NP decreased, and the bond length increased. Except for rutin, the optimal binding sites between NP and other model drugs shifted. These changes suggest that the competitive binding of PEK hinders the intermolecular hydrogen bonding between the drugs and NP, reducing their affinity with NP and thereby allowing more drugs to diffuse downward in molecular form. This finding offers an additional perspective on how PEK can promote the transdermal permeability of drugs.

#### 3.4.6. Molecular Dynamics Simulation

**Figure 16:**
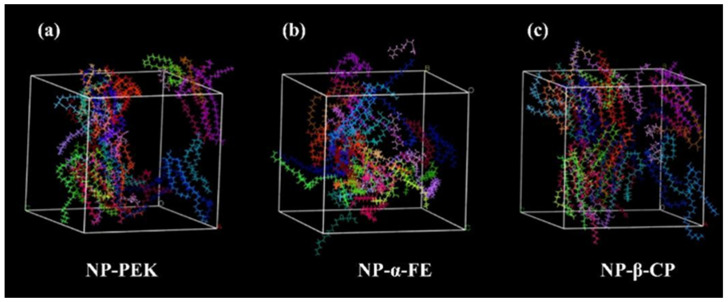
Snapshots of the simulated systems at the end stage of the MS. (**a**) PEK in NP box. (**b**) α-FE in NP box. (**c**) β-CP in NP box. (NP: ceramide NP; PEK: perilla ketone; β-CP: β-caryophyllene; α-FE: (*Z*,*E*)-α-farnesene).

MS was used to analyze the ability of PEs to promote transdermal drug absorption by calculating the kinetic parameters of their interaction with lipid membranes. The *CED* value was used to quantify the strength of intermolecular interactions within the simulated system, while the *D′* value represented the dynamic changes in the movement of PEs within the membrane [60]. In this study, the *CED* and *D′* values for three compounds with concentrations exceeding 1% in PO within NP boxes were calculated, as shown in Figure 16. In the simulation system, the *CED* values of PEK, β-CP, and α-FE were 9.68 × 107, 8.41 × 107, and 8.07 × 107 kcal·mol^−1^, respectively, indicating the strongest intermolecular interaction between PEK and NP. The *D′* values, obtained by fitting MSD curves, were 4.629 × 10^−2^, 6.914 × 10^−2^, and 6.353 × 10^−2^ Å·ps^−1^, respectively, suggesting that the diffusion rate of PEK in the lipid membrane was the lowest. This slower diffusion rate implies that PEK can remain in the SC for a longer period of time. According to a previous study [61], prolonging the retention time of PEs in the SC enhances their penetration enhancement effect. The MS data from this study further support the reliability of using PEK instead of PO from a dynamic perspective. The penetration-enhancing effect of PO might be weaker than that of pure PEK due to inactive compounds in the essential oil. The essential oil’s penetration-enhancing efficacy is predominantly PEK-driven. To achieve effects comparable to pure PEK, higher doses were required in the formulations.

#### 3.4.7. Transepidermal Water Loss Experiment

**Figure 17:**
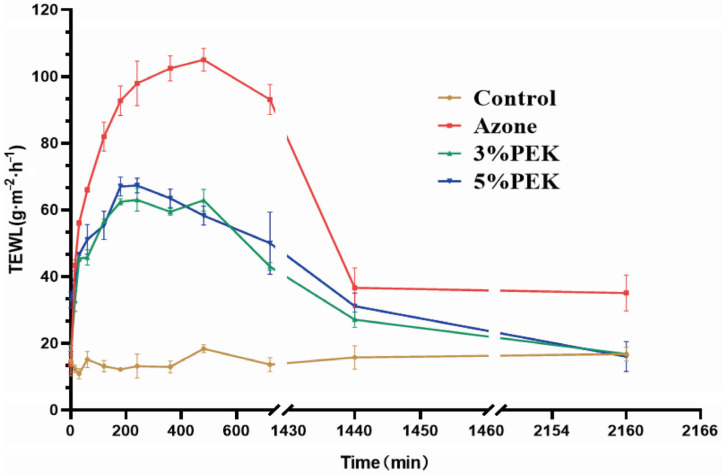
TEWL of the rat skin after treatment with different penetration enhancers (*n* = 4, PEK: perilla ketone; TEWL: transepidermal water loss).

The moisture loss from the surface of the skin is closely linked to its barrier function, and the extent of this loss serves as an indicator of changes in skin barrier integrity [62]. The variations in TEWL are depicted in Figure 17. Throughout the experiment, the TEWL values in the control group remained consistent across all the detection time points. However, after the administration of penetration enhancers for 5 min, the TEWL values of the 3% (*w*/*v*) PEK group, 5% (*w*/*v*) PEK group, and azone group significantly increased compared with those of the control group (*p* < 0.05). This indicates that the application of penetration enhancers can rapidly impair the skin’s barrier function, leading to an increase in water loss from the skin.

The maximum TEWL values in the PEK groups occurred at the fourth hour after the start of the experiment, with the TEWL values in the low- and high-dose PEK groups being 63.00 ± 4.81 and 67.30 ± 3.11 g·cm^−2^·h^−1^, respectively, showing significant differences compared with the control group (*p* < 0.05). The TEWL values at 24 h were significantly lower than the peak values (*p* < 0.05) and returned to normal levels after 36 h (*p* > 0.05). In the azone group, the increase in TEWL values slowed after 4 h and reached its peak at 8 h (97.95 ± 9.40 g·cm^−2^ · h^−1^). The TEWL values gradually decreased after 24 h and did not return to normal until the end of the 36 h experiment. These results indicate that PEK can effectively reduce the skin’s barrier function, with a reversible effect similar to that of other terpenoid penetration enhancers [63]. The TEWL test results are consistent with those of the skin irritation test, further demonstrating that PEK is safer than azone.

## 4. Conclusions

In this study, the essential oil of *P. frutescens* was extracted and subjected to GC-MS and ^1^H NMR analysis. The principal active component identified in the essential oil was PEK, which demonstrated the ability to enhance the skin permeation of five model drugs with minimal skin irritation. The mechanism underlying PEK’s penetration-enhancing effect was explored in detail. It was found that PEK facilitates the distribution of drugs from their solution phase into the SC. Furthermore, PEK interacts with the lipid bilayer of the skin by forming competitive intermolecular H-bonds with the amide groups located in the hydrophilic heads of intercellular lipid molecules. These interactions reduce the affinity between drugs and lipids, disrupting the organized hydrogen bonding network within the lipid bilayers. This disruption leads to an increase in the fluidity of intercellular lipids, which ultimately lowers the resistance to drug diffusion across the SC. The results of the TEWL test further demonstrated that PEK’s effects on skin permeability are reversible. Notably, PEK exhibited penetration enhancement that was both effective and transient, a characteristic similar to that of other terpenoid penetration enhancers, which suggests its potential for controlled use in drug delivery systems. In conclusion, this study introduces PEK as a promising, low-irritant terpenoid compound that can significantly enhance the transdermal diffusion of drugs, especially those with high polarity and strong hydrogen bonding capacity. PEK accelerates the diffusion of such drugs within the SC by improving the fluidity of the lipid bilayer and reducing the barriers to drug diffusion. However, it is essential to consider the physicochemical properties of the drug when selecting suitable candidates for transdermal delivery, as these properties are crucial for optimizing cumulative transdermal permeability.

## Figures and Tables

**Table 1:** Physicochemical properties of the model drugs.

Model Drug	M.W. (Daltons)	M.P. (°C)	Log *P*	PSA (Å^2^)	Hydrogen Bond Acceptors	Hydrogen Bond Donors
Ferulic acid	194.18	168–172	1.64	66.76	4	2
Rutin	610.52	195	−0.87	269.43	16	10
Paeoniflorin	480.46	124	−0.39	164.37	11	5
Puerarin	416.38	187–189	0.48	160.82	9	6
Luteolin	286.23	330	2.4	111.13	6	4

M.W.: molecular weight; M.P.: melting point; log *P*: oil–water partition coefficient; PSA: polar surface area. All the data were obtained from the following website: https://www.chemsrc.com (accessed on 17 October 2024).

**Table 2:** Parameters of the model drugs’ permeation through the skin with or without PEK (mean ± SD, *n* = 4).

Model Drug	Penetration Enhancer	*T*_lag_ (h)	*C* (μg·mL^−1^)	*J*_ss_ (μg·cm^−2^·h^−1^)	*Q*_8h_ (μg·cm^−2^)	*QER*
Ferulic acid	Control	2.40 ± 0.15	307.06 ± 10.12	14.79 ± 0.77	83.50 ± 5.48	1
	3% PEK	1.81 ± 0.23	467.53 ± 20.31	28.15 ± 5.29 **	139.45 ± 7.93 *	1.67 ± 0.10
	5% PEK	2.01 ± 0.21	546.45 ± 29.57	31.06 ± 5.64 **	154.48 ± 10.56 **	1.85 ± 0.13
Rutin	Control	1.47 ± 0.69	29.82 ± 3.05	0.60 ± 0.04	3.69 ± 0.51	1.00
	3% PEK	2.43 ± 0.3	37.55 ± 5.63	1.67 ± 0.20 *	10.70 ± 1.23 *	2.90 ± 0.33
	5% PEK	2.19 ± 0.17	35.61 ± 4.42	1.82 ± 0.12 **	11.51 ± 0.62 **	3.12 ± 0.17
Paeoniflorin	Control	1.32 ± 0.46	62.42 ± 5.70	2.07 ± 0.05	14.05 ± 1.10	1
	3% PEK	1.60 ± 0.29	72.93 ± 6.89	3.99 ± 0.87 *	31.05 ± 4.25 *	2.21 ± 0.30
	5% PEK	1.49 ± 0.33	99.09 ± 8.15	5.98 ± 0.43 **	34.56 ± 3.11 **	2.46 ± 0.22
Puerarin	Control	2.29 ± 0.32	875.52 ± 7.56	4.17 ± 0.45	23.58 ± 1.43	1
	3% PEK	1.22 ± 0.2	948.70 ± 5.68	13.50 ± 0.05 **	69.80 ± 1.68 **	2.96 ± 0.07
	5% PEK	1.84 ± 0.29	1138.95 ± 10.87	16.64 ± 0.64 **	79.94 ± 4.90 **	3.39 ± 0.21
Luteolin	Control	0.94 ± 0.19	38.84 ± 1.05	0.87 ± 0.01	1.72 ± 0.03	1
	3% PEK	1.77 ± 0.43	36.03 ± 2.13	1.26 ± 0.08 **	4.35 ± 0.32 **	2.53 ± 0.19
	5% PEK	1.71 ± 0.44	40.11 ± 3.95	2.39 ± 0.07 **	4.40 ± 0.26 **	2.56 ± 0.15

* *p* < 0.05, ** *p* < 0.01 compared to the control group. *T*_lag_: lag time; *C*: drug concentration; *J*_ss_: steady-state flux; *Q*_8h_: cumulative amount of the drug permeated at 8 h; *QER*: quantitative enhancement ratio.

**Table 3:** Solubility parameters of the chemicals.

Model Drug	*δ* (J·cm^−3^)^1/2^	Δ*δ* (J·cm^−3^)^1/2^
NP	18.68	—
PEK	18.1	−0.58
Ferulic acid	23.54	4.86
Rutin	43.74	25.06
Paeoniflorin	25.02	6.34
Puerarin	45.34	26.66
Luteolin	43.82	25.14

NP: ceramide NP; PEK: perilla ketone; *δ*: solubility parameter; Δ*δ*: the difference in solubility parameters.

**Table 4:** Peak area of ν_as_CH_2_ and ν_s_CH_2_ stretching absorbance with or without enhancers and their percentage decreases.

Sample	ν_as_CH_2_		ν_s_CH_2_	
	Peak Area	Decrease in Peak Area ^a^ (%)	Peak Area	Decrease in Peak Area ^a^ (%)
Blank	106.28	—	70.54	—
3% PEK	104.69	1.50	69.22	1.87
5% PEK	103.76	2.37	69.10	2.04

^a^ Percentage decrease in peak area: peak area with PEK/peak area in control × 100%. ν_as_CH_2_: CH_2_ asymmetric stretching vibrations; ν_s_CH_2_: symmetric stretching vibrations; PEK: perilla ketone.

## Data Availability

The data presented in this paper can be made available upon request to the corresponding author.

## Supplementary Materials

The following supporting information can be downloaded at: https://www.mdpi.com/article/10.3390/pharmaceutics17020254/s1, Figure S1: GC chromatogram of perilla essential oil. (a) GC chromatogram of perilla essential oil with the main component removed to facilitate the integration of other components. (b) GC chromatogram of perilla essential oil with proper dilution to facilitate the detection of the main component. Table S1: Chromatographic conditions of the model drugs; Table S2: Components of perilla essential oil determined by GC-MS; Table S3: SC/solution partition coefficient of the model drugs (mean ± SD, *n* = 4); Table S4: Permeability coefficient of the model drugs (mean ± SD, *n* = 4).
