# Supplementary material for: A Novel Natural Penetration Enhancer for Transdermal Drug Delivery: In Vitro/In Vivo Evaluation and Penetration Enhancement Mechanism
Source: Pharmaceutics. 2025 Feb 14;17(2):254. doi: 10.3390/pharmaceutics17020254 (PMC11859311; doi:10.3390/pharmaceutics17020254)
Supplement: Supplementary file 1 [file pharmaceutics-17-00254-s001.zip › pharmaceutics-3442907-supplementary.pdf]

Supporting Information

A Novel Natural Penetration Enhancer for Transdermal Drug Delivery: In Vitro/In Vivo Evaluation and Penetration Enhancement Mechanism

Nanxi Zhao [1,†], Jiale Hao [1,†], Yucong Zhao [1], Bingqian Zhao [1], Jiayu Lin [2], Jian Song [1], Manli Wang [1,*] and Zheng Luo [1,*]

[1] Department of Pharmaceutical Sciences, College of Pharmacy, Beihua University, Jilin 132013, China

[2] Department of Pathology, Jilin Central Hospital, Jilin 132013, China

* Correspondence: luozhengspu@163.com (Z.L.); bhwangml@163.com (M.W.)

† These authors contributed equally to this work

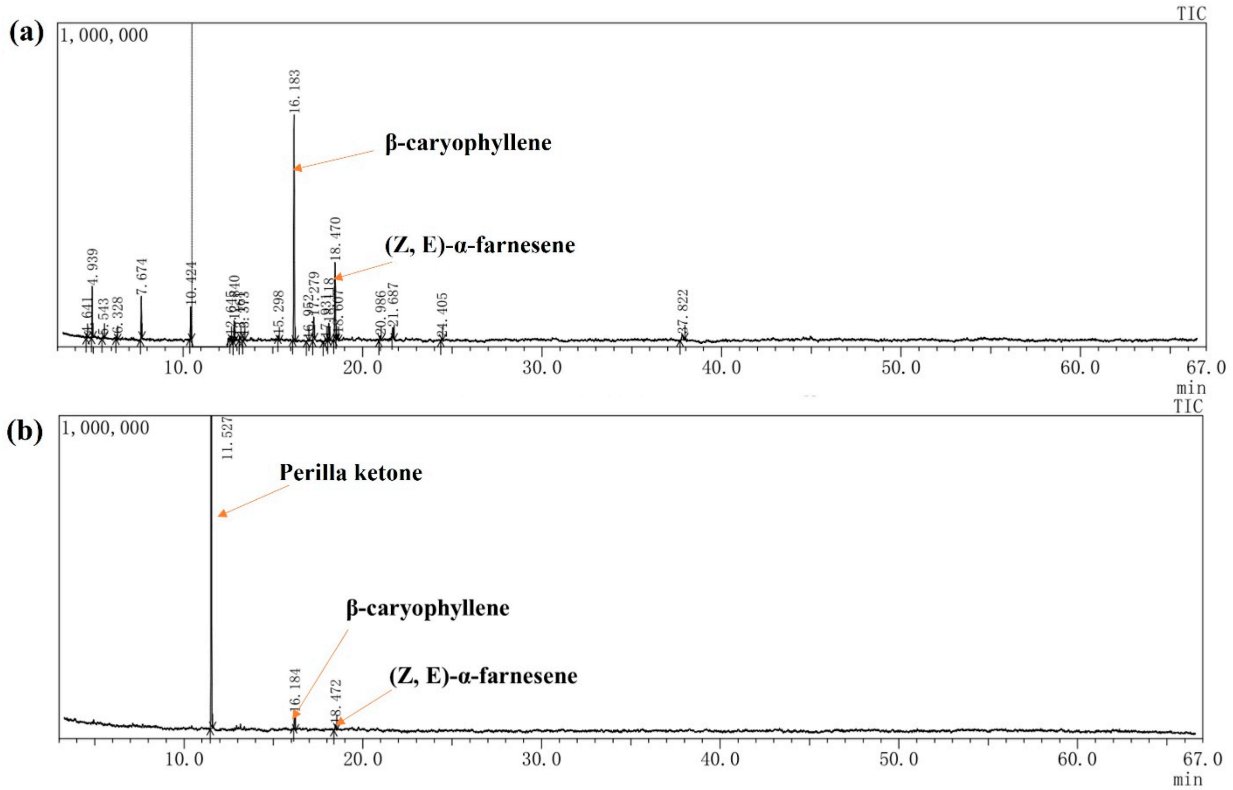

Figure S1. GC chromatogram of perilla essential oil. (**a**) GC chromatogram of perilla essential oil with the main component removed to facilitate the integration of other components. (**b**) GC chromatogram of perilla essential oil with proper dilution to facilitate the detection of the main component.

Table S1 Chromatographic conditions of the model drugs.

Model Drugs	Mobile Phase (v/v)	Temperature (°C)	Wave Length (nm)	Standard Curve	Linear Range (μg·mL^{-1})
Ferulic Acid	ACN:0.1%H_3PO_4 (25:75)	30	321	y = 66743x - 70531 R^2 = 0.9995	0.8-160.0
Paeoniflorin	ACN:0.1%H_3PO_4 (20:80)	30	232	y = 15741x + 9222.4 R^2 = 0.9998	4.0-160.0
Puerarin	ACN:0.1%H_3PO_4 (15:85)	40	250	y = 12950x + 11089 R^2 = 0.9995	4.0-160.0
Luteolin	ACN:0.1%H_3PO_4 (30:70)	30	350	y = 59764x - 4968.1 R^2 = 0.9998	0.09-3.0
Rutin	MeOH:0.1%H_3PO_4 (55:45)	30	340	y = 13686x - 295.46 R^2 = 0.9999	0.1-4.0

Table S2 Components of perilla essential oil determined by GC-MS.

No.	Retention Time	Component	Area Ratio (%)
1	4.64	Benzaldehyde	0.03
2	4.94	1-Octen-3-ol	0.38
3	5.54	3,3-Dimethyloctane	0.01
4	6.33	Benzeneacetaldehyde	0.04
5	7.67	Linalool	0.36
6	10.42	2-Pentanonefd cxs	0.30
7	11.46	Perilla ketone	93.70
8	12.65	Egomaketone	0.04
9	12.84	Isoegomaketone	0.19
10	13.16	7-Methyl-1-undecene	0.04
11	13.37	11-Methyldodecanol	0.01
12	15.30	Cyclohexane	0.03
13	16.18	β-caryophyllene	2.30
14	16.95	2-Methyl-2-trimethylsilyl-trans-hexahydro-1,3-benzodithiane	0.01
15	17.28	Humulene	0.29
16	17.93	Digitoxin	0.05
17	18.12	Germacrene D	0.23
18	18.47	(Z, E)-α-farnesene	1.03
19	18.61	γ-Elemene	0.01
20	20.99	Nerolidol	0.08
21	21.69	Caryophyllene oxide	0.12
22	24.41	5-Chlorovaleric acid	0.02
23	37.82	Dibutyl phthalate	0.10

Table S3 SC/solution partition coefficient of the model drugs (mean ± SD, n = 4).

Model Drugs	Penetration Enhancers	K (×10^{-2})	KER
Ferulic acid	Control	7.71±0.32	1
	3% PEK	9.71±0.47*	1.26±0.06
	5% PEK	12.49±1.58*#	1.62±0.20
Rutin	Control	4.15±0.37	1
	3% PEK	14.69±1.88**	3.54±0.45
	5% PEK	15.60±1.26**	3.76±0.30
Paeoniflorin	Control	4.39±0.71	1
	3% PEK	10.10±0.96**	2.30±0.22
	5% PEK	10.71±0.65**#	2.44±0.15
Puerarin	Control	11.11±0.29	1
	3% PEK	31.89±2.31**	2.87±0.21
	5% PEK	34.55±2.15**	3.11±0.19
Luteolin	Control	7.30±0.63	1
	3% PEK	14.97±0.68*	2.05±0.09
	5% PEK	20.22±1.47**	2.77±0.20

* $p < 0.05$, ** $p < 0.01$ compared to the control group.

Table S4 Permeability coefficient of the model drugs (mean ± SD, n = 4).

Model Drugs	Penetration Enhancers	P (cm ·h^{-1} ·10^{-3})	PER
Ferulic acid	Control	48.17±2.51	1
	3% PEK	60.21±11.31*	1.25±0.23
	5% PEK	56.84±10.32*	1.18±0.21
Rutin	Control	20.12±1.34	1
	3% PEK	44.47±5.33**	2.21±0.26
	5% PEK	51.10±3.37**	2.54±0.17
Paeoniflorin	Control	33.16±0.8	1
	3% PEK	54.71±11.93**	1.65±0.36
	5% PEK	60.35±7.37**	1.82±0.22
Puerarin	Control	4.76±0.51	1
	3% PEK	14.23±0.05**	2.99±0.01
	5% PEK	14.61±0.56**	3.07±0.12
Luteolin	Control	22.40±0.26	1
	3% PEK	34.97±2.22*	2.56±0.10
	5% PEK	59.58±3.75**	2.66±0.17

* $p < 0.05$, ** $p < 0.01$ compared to the control group.
